# Revising CX3CR1 Expression on Murine Classical and Non-classical Monocytes

**DOI:** 10.3389/fimmu.2020.01117

**Published:** 2020-06-03

**Authors:** Aïda Meghraoui-Kheddar, Sandrine Barthelemy, Alexandre Boissonnas, Christophe Combadière

**Affiliations:** ^1^Sorbonne Université, Inserm, CNRS, Centre d'Immunologie et des Maladies Infectieuses, Cimi-Paris, Paris, France; ^2^Université Côte d'Azur, CNRS UMR7275, Institut de Pharmacologie Moléculaire et Cellulaire (IPMC), Valbonne, France

**Keywords:** monocytes, chemokine receptor, CX3CR1, CD43, multiparametric analysis

## Abstract

In mice, monocytes (Mo) are conventionally described as CX3CR1^low^ classical Mo (CMo) and CX3CR1^high^ non-classical Mo (NCMo) based on the expression of EGFP in Cx3cr1^+/*EGFP*^ mice and by analogy with human CX3CR1 expression. Although this terminology is widely used, it may not reflect the expression of CX3CR1 on Mo subsets. Using an unsupervised multiparametric analysis of blood Mo in steady state and after sterile peritonitis, we observed that CX3CR1 expression did not discriminate the CMo from the NCMo subsets. Our results highlight that despite being a reliable reporter to discriminate Mo subpopulations, EGFP level in Cx3cr1^+/*EGFP*^ mice does not reflect CX3CR1 expression measured by a fluorescently-labeled CX3CL1 chemokine and a CX3CR1 specific antibody. In conclusion, authors should be cautious not to identify murine classical and non-classical Mo as CX3CR1^low^ and CX3CR1^high^ but rather use alternative markers such as the combination of Ly6C and CD43.

## Introduction

Chemokine receptors are key G protein-coupled receptors for immune cell trafficking in inflammation and physiological conditions. They are critical for lymphocytes homing, for normal lymphoid tissue development, for Mo egress from bone marrow (BM) and facilitate organ infiltration of immune cells (1). Because of their selective expression on leukocyte subsets, they are useful cell surface markers that identify immune cell subtypes like CCR7 for naïve T cells, CXCR5 for T follicular helper lymphocytes, CCR5 and CXCR3 for type 1 lymphocytes or CCR2 and CX3CR1 to discriminate Mo subsets. The latter initially described as an orphan seven-transmembrane domain receptor named alternatively CMKBRL1 (2, 3) or V28 (4) is the specific high-affinity and functional receptor for the chemokine CX3CL1 in mice and human (2, 3, 5, 6). This chemokine is the sole member of the CX3C chemokine subfamily and was identified in human cells as Fractalkine (7) and in mouse activated brain microglia as neurotactin (8). It was characterized as a versatile molecule that directed migration of Mo, NK, and T cells by its soluble form and regulates adhesion of these cells by its membrane-bound form expressed on endothelial cells (5).

In 2000, Jung et al. (9) generated a transgenic mouse strain, where the *Cx3cr1* gene was replaced with the gene encoding the enhanced green fluorescent protein (EGFP) and analyzed it in heterozygote (*Cx3cr1*^+/*EGFP*^) or homozygote (*Cx3cr1*^*EGFP*/*EGFP*^) configuration. This approach allowed the examination of the CX3CR1 expression pattern and migration of cells that normally express this receptor. Based on the green fluorescence and the use of a Fractalkine/NTN-Fc fusion peptide, they confirmed the presence of CX3CR1 on the surface of Mo, part of NK cells, circulating and skin resident DC and microglia. However, the CX3CL1 receptor was absent in resting tissue macrophages (hepatic Kupffer cells, splenic, and peritoneal macrophages), astrocytes, and oligodendrocytes, neutrophils and eosinophils, B lymphocytes, resting and concanavalin A activated T cells, unlike what has been observed in humans. Nonetheless, recent works clearly demonstrated that terminally differentiated cytotoxic CD8^+^ T cells express CX3CR1 (10). Consequently to Jung work, *Cx3cr1*^+/*EGFP*^ and *Cx3cr1*^*EGFP*/*EGFP*^ mice have become widely used and EGFP fluorescence level was used to monitor CX3CR1 expression in several cell populations and its modulation through time and under several pathological conditions (e.g., inflammation, infection, cancer) (11–15).

In mice, Mo are differentiated in two subsets. It was first achieved on their expression of CCR2, CD62L, and CX3CR1 measured by expression of EGFP in cells from *Cx3cr1*^+/*EGFP*^ mice (16). One Mo subset express CCR2, CD62L, and only moderate amounts of EGFP and are known as the 'inflammatory' subset, whereas the second that does not express CCR2 or CD62L but display higher expression of EGFP and CD43 is referred as patrolling. In addition, Geissmann et al. (17) identified Ly6C as an additional marker of inflammatory Mo in mice. These studies indicated that CCR2^+^CD62L^+^CX3CR1-EGFP^low^Ly6C^+^ mouse Mo correspond to CD14^hi^CD16^−^ classical human Mo, which are also CCR2^+^CX3CR1^low^ and that CCR2^−^CD62L^−^CX3CR1-EGFP^hi^Ly6C^low^ mouse Mo correspond to CD14^low^CD16^+^ human non-classical Mo, which also express large amounts of CX3CR1. These observations were the first to indicate that it would be possible to address the *in vivo* relevance of human Mo heterogeneity by studying mice.

So far, the level of expression of EGFP combined to the detection of Ly6C (or Gr1) marker in *Cx3cr1*^+/*EGFP*^ mice was the most often applied strategy to differentiate CMo, assumed as Ly6C^high^CX3CR1^low^, from NCMo, assumed as Ly6C^low^CX3CR1^high^ (18). This strategy was and still is commonly used, despite the fact that not all green fluorescent cells in *Cx3cr1*^+/*EGFP*^ mice would be expected to be CX3CR1^+^. Cells that ceased to express the CX3CR1 are likely to harbor residual EGFP because of the extended half-life of the EGFP protein (>24 h) (9). Green fluorescence in these cells would thus indicate their derivation from CX3CR1-expressing cells but may not reflect the cell expression of the receptor. In fact, Hamon at al. (19) observed in *Cx3cr1*^+/*EGFP*^ mice that while the EGFP fluorescent intensity was significantly higher in circulating Ly6C^low^ Mo than Ly6C^high^ Mo, staining with fluorescently-labeled CX3CL1 showed an equivalent level of binding. This discrepancy was also observed in the bone marrow. Here, we addressed the expression of CX3CR1 on murine Mo in homeostatic and inflammatory conditions using both its specific ligand and antibody.

## Materials and Methods

### Mice

C57Bl6 mice were purchased from Elevage Janvier (Le Genest, Saint Isle, France). *Cx3cr1*^+/*EGFP*^, *Cx3cr1*^*EGFP*/*EGFP*^ (9) and *Nr4a1*^+/*EGFP*^ (20) mice were bred in our animal facility “UMS 028—Phénotypage du petit animal.” All experiments' protocols were approved by the local ethic committee.

### Sterile Peritonitis Model

LPS was administered intraperitoneally at 300 ng/kg in 100 μl phosphate-buffered saline (PBS). LPS-free PBS was administered for control group.

### Blood Tissue Partitioning

Intravascular (i.v.) CD45 labeling was performed, as previously described (19). Mice were injected i.v. with 2 μg of anti-CD45 (clone 30-F11) in PBS. Two minutes after injection, blood was drawn and mice were sacrificed. Lungs and spleen were immediately harvested and bathed in a large volume of PBS and bone marrow (BM) cells were harvested by flushing out the thighbone with PBS. CD45-labeled cells in all tissues were considered to be intravascular and CD45^−^ cells were considered to be parenchymal.

### Cell Preparation

Blood was drawn via retro-orbital puncture with heparin and directly stained with antibodies.

The spleen and the lung were digested in RPMI-1640 medium (Gibco, ThermoFisher, Illkirch, France) containing 1 mg/mL collagenase IV (Sigma Aldrich, Merck, St. Quentin Fallavier) for 30 min at 37°C. After digestion, tissues were mashed through a 30 μm pore cell strainer (Miltenyi Biotec, Bergisch Gladbach, Germany) and washed in PBS. Cell surface staining was performed by incubating for 20 min the freshly prepared cells or whole blood with Panel-1 antibodies (Supplemental Table 1). After staining, erythrocytes from the blood were lysed using Pharm Lyse Buffer (BD, Le Pont de Claix, France) and tissue-cell suspensions were washed once using PBS and analyzed thereafter by flow cytometry.

For surface CX3CR1 staining on circulating monocytes, lysed blood cells were incubated or not with 50, 100, or 200 nM murine CX3CL1-AF647 (Almac, Edinburgh, Scotland) for 30 min at 37°C. Cell were washed once in PBS and cell surface staining was performed using Panel-2 (Supplemental Table 1). When circulating Mo were not incubated with CX3CL1-AF647, CX3CR1 was detected using Anti-CX3CR1-PE. Specificity of CX3CR1 staining was controlled using either CX3CR1-deficient Mo isolated from *Cx3cr1*^*EGFP*/*EGFP*^ mice or incubating CX3CR1-proficient Mo in the presence of unlabeled CX3CL1 at 1μM before staining.

Sample acquisitions were performed on the LSRFortessa X-20 Flow cytometry (BD) using FACSDIVA software (BD), and data were analyzed with FlowJo software (BD) and Cytobank analysis platform (21) (Beckman Coulter, Santa Clara, CA).

### Data Presentation and Statistical Analysis

Mean ± SD are presented for all quantifications. Nonparametric two-tailed Mann–Whitney test with a significance threshold of alpha (α = 0.05) was used to compare differences in mean fluorescence intensity (MFI) between two groups. Nonparametric two-tailed Wilcoxon signed-rank test with a significance threshold of alpha (α = 0.05) was used to compare differences in MFI from two matched samples. Statistical tests were performed using commercial statistics software Prism (GraphPad, San Diego, CA).

## Results and Discussion

### CD43 but Not CX3CR1 Surface Expression Discriminates Mo Subsets

**Figure 1:**
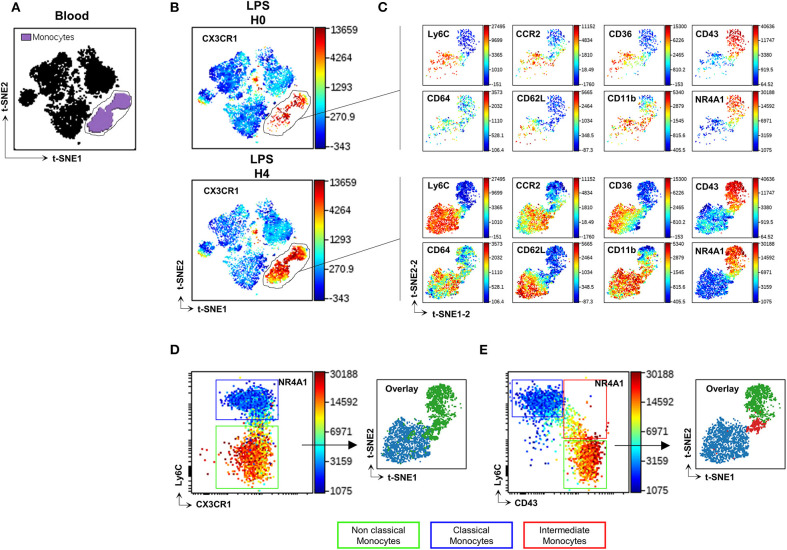
CD43 but not CX3CR1 surface expression discriminates Mo subsets. t-SNE was used to automatically arrange circulating immune cells according to their expression profile of 16 proteins before (H0) and after (H4) LPS injection in a 2D t-SNE1/t-SNE2 plot **(A)**. The expression of CX3CR1 was presented in a color scale going from blue to red **(B)**. Mo (gated purple cluster in panel **A**) were embedded in a new set of t-SNE axes designated t-SNE1-2 and t-SNE2-2 and the expression of Ly6C, CD64, CCR2, CD36, CD11b, CD43 markers, and *Nr4a1* reporter were presented at H0 and H4 after LPS injection in a color scale going from blue to red **(C)**. Dot plots compare the relative surface expression of CX3CR1 **(D)** and CD43 **(E)** on Mo to discriminate the subsets. Each subset was back viewed and overlaid on the t-SNE1-2/t-SNE2-2 plot. Dot-plots represent a merged file of three mice per group.

Blood Mo were characterized using an unsupervised analysis based on previously described markers (16, 17) and listed in Supplemental Table 1 as Panel-1. The Visualization of t-Distributed Stochastic Neighbour Embedding (viSNE implementation of t-SNE) (22) was used to automatically arrange circulating immune cells according to their expression profile of the Panel-1 proteins before and after LPS injection. Position on the 2D map represents local phenotypic similarity (Figure 1). Circulating Mo (purple gate, Figure 1A) were gated apart from the other circulating immune cells (black cluster, Figure 1A) on the viSNE map, based on the relative expression of CD11b, Ly6C, CCR2, CD43, CD36, CD64, *Nr4a1* reporter, and CX3CR1 (Figure 1B and Supplemental Figure 1). A second t-SNE map was generated on the former and showed good discrimination of two dominant clusters identified as CMo with preferential expression of Ly6C, CD64, CCR2, CD62L, CD36, CD11b proteins, and NCMo with selective expression of CD43 protein and the orphan nuclear receptor Nuclear Receptor Subfamily 4 Group A Member 1 (NR4A1), a transcription factor involved in NCMo differentiation and survival (23), monitored by EGFP reporter from the *Nr4a1*^+/*EGFP*^ transgenic mouse (Figure 1C, Supplemental Figure 2). The unsupervised analysis uncovered that surface CX3CR1 expression was homogenous across all Mo subsets contrariwise to the expected phenotype of CMo and NCMo as CX3CR1^low^ and ^high^, respectively (Figures 1B,D). Discriminating Mo subsets using Ly6C and CD43 markers allowed a better identification of CMo (blue gate and blue cluster, Figure 1E) and NCMo (green gate and green cluster) with a better definition of the intermediate Mo (IMo) subsets (red gate and red cluster).

### CMo Express Higher Level of CX3CR1 at the Membrane and Uptake More Soluble Ligand Than NCMo

**Figure 2:**
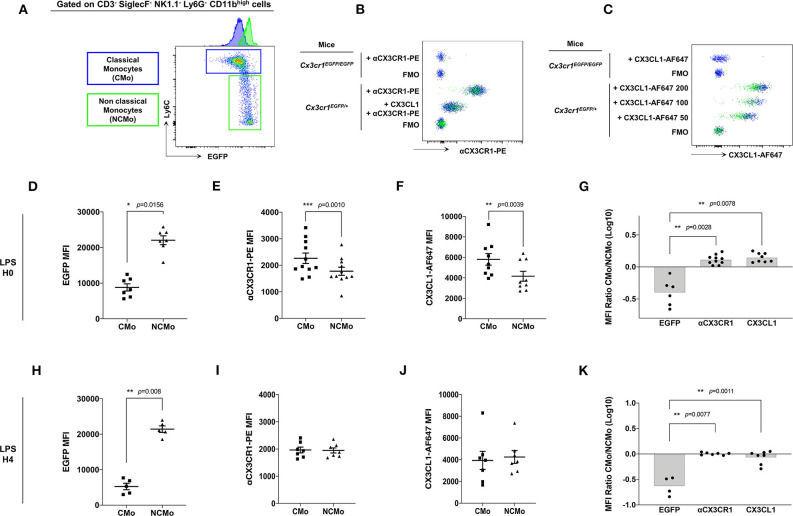
CMo express higher level of CX3CR1 at the membrane and uptake more soluble ligand than NCMo. In *Cx3cr1*^+/*EGFP*^ mice, classical (CMo, blue gate) and non-classical (NCMo, green gate) circulating monocytes were gated on the basis of Ly6C and EGFP expression level as CX3CR1-EGFP^low^ Ly6C^high^ and CX3CR1-EGFP^high^ Ly6C^low^ cells, respectively, **(A)**. CX3CR1 expression level was assessed using anti-CX3CR1-PE antibody whose specificity was confirmed by competition with CX3CL1 **(B)**, and using CX3CL1-AF647 at indicated concentration expressed in nM **(C)**. Staining and binding specificity were assessed using a full minus-one stained sample (FMO) and Mo isolated from *Cx3cr1*^*EGFP*/*EGFP*^ mice. MFI of EGFP [**(D)** *n* = 7; **(H)** *n* = 5], anti-CX3CR1-PE antibody [**(E)** *n* = 11; **(I)** *n* = 7] and CX3CL1-AF647 [**(F)** *n* = 9; **(J)** *n* = 7] were measured on circulating classical (CMo) and non-classical (NCMo) subsets in *Cx3cr1*^+/*EGFP*^ mice at H0 **(D–F)** and 4 h after LPS intra-peritoneal injections **(H–J)**. Non-parametric two-tailed Wilcoxon signed-rank test was used to compare differences in MFI from two matched samples. Ratio of CMo MFI over NCMo MFI were calculated at H0 [**(G)** *n* = 6–10] and H4 [**(K)** *n* = 4–7] after LPS injection. *p < 0.05, **p < 0.01, ***p < 0.001.

CX3CR1 expression on murine Mo was previously evaluated mainly on EGFP fluorescent reporter of the *Cx3cr1*^*EGFP*/+^ knock-in mice leading to the consensual nomenclature presenting CMo (blue gate, Figure 2A) as CX3CR1^low^ and NCMo (green gate, Figure 2A) as CX3CR1^high^. Here, we investigated whether EGFP expression correlated with CX3CR1 surface expression on both CMo and NCMo. We first validated that the monoclonal anti-CX3CR1-PE (clone: SA011F11) provides a reliable staining of CX3CR1 in *Cx3cr1*^+/EGFP^ compared to circulating Mo from *Cx3cr1*^EGFP/EGFP^ mice (Figure 2B) and further evaluated the functional efficacy of this receptor to bind and uptake its cognate ligand using fluorescently tagged CX3CL1 (Figure 2C).

As historically described (9), Ly6C^high^ CMo from *Cx3cr1*^+/*EGFP*^ mice, expressed a significantly lower amount of EGFP compared to Ly6C^low^ NCMo (MFI = 8,805 ± 2,652, MFI = 22,056 ± 3,266, respectively, *p* = 0.0156; Figure 2D). However, CMo expressed a higher level of CX3CR1, as it is measured either with anti-CX3CR1-PE antibody staining (MFI = 2,265 ± 648, MFI = 1,777 ± 500, respectively, *p* = 0.0010; Figure 2E) or with CX3CL1-AF647 uptake (MFI = 5,808 ± 1,694, MFI = 4,164 ± 1,447, respectively, *p* = 0.0039; Figure 2F). Based on these results Ly6C^high^ CMo were CX3CR1^+^ and EGFP^low^ whereas Ly6C^low^ NCMo were CX3CR1^+^ and EGFP^high^ with slightly lower anti-CX3CR1-PE and CX3CL1-A647 MFI in the latter. The ratio of CX3CR1 MFI between CMo and NCMo clearly indicates the discordance between the indirect measure of CX3CR1 using the cytosolic EGFP reporter and the direct measure of CX3CR1 cell surface expression based on antibody binding and soluble ligand avidity (Figure 2G). Similar discrepancy was observed on circulating CMo and NCMo before (Figures 2D–G) and 4 h after intra-peritoneal LPS injection (Figures 2H–K), indicating that, in both homeostatic and certain inflammatory conditions, CMo display higher level of functional CX3CR1 than NCMo, challenging the consensual definition of Mo subsets in mice.

### CD43 Expression Identifies Circulating and Tissue Resident NCMo Subsets

**Figure 3:**
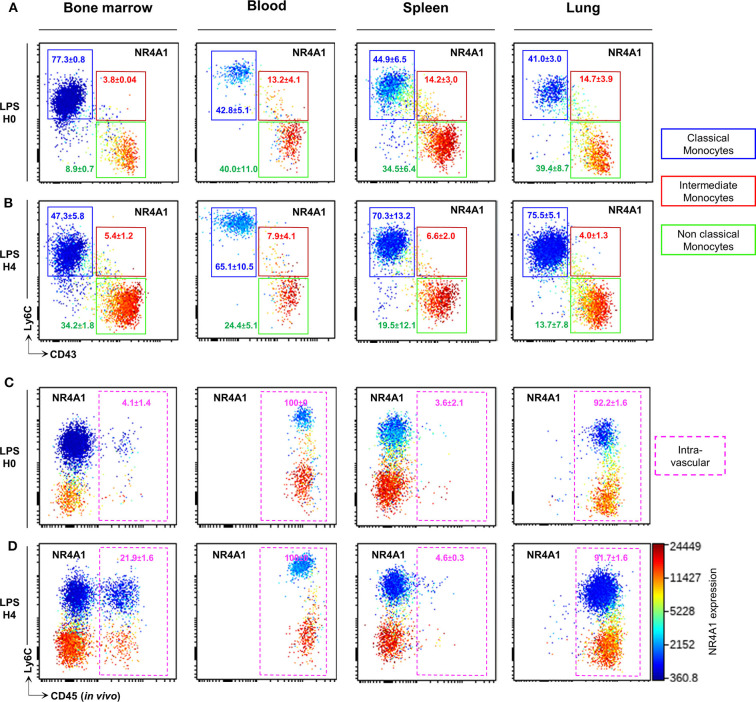
CD43 expression identifies circulating and tissue resident NCMo subsets. Classical (blue gate), intermediate (red gate), and non-classial (green gate) monocyte subsets were gated according to Ly6C and CD43 markers expression in the bone marrow, the blood, the spleen, and the lungs before **(A)** and 4 h after LPS intraperitoneal injection **(B)**. The distribution of monocytes subsets between the vasculature and the organs parenchyma was evaluated by blood tissue partitioning (intravascular CD45 staining) before **(C)** and 4 h after LPS intraperitoneal injection **(D)**. *Nr4a1* reporter expression (using *Nr4a1*^+/*EGFP*^ mouse was presented in each dot plot in a color scale going from blue to red **(A–D)**. Dot-plots are representative of *n* = 3 per group.

We next evaluated whether the combination of CD43 and Ly6C surface markers that allow unambiguous identification of blood CMo from NCMo and IMo, would be efficient in other tissues using Panel-1 (Supplemental Table 1). Mo were harvested from the bone marrow, the spleen, and the lungs after blood tissue partitioning by i.v. injection of a fluorescently labeled anti-CD45 to identify vascular resident cells (CD45^+^) from tissue-resident cells (CD45^−^) (24) at homeostasis and 4h after LPS intraperitoneal injection. As observed in the blood, CD43 clearly identify all myeloid cells expressing NRR4A1, in all the tested organs and, in homeostatic (Figure 3A) and inflammatory conditions (Figure 3B). Blood tissue partitioning revealed that most of the lung NCMo and CMo reside in the vasculature [92.2 ± 1.6% CD45^+^ cells before (Figure 3C) and 91.7 ± 1.6 CD45^+^ 4 h after LPS injection (Figure 3D)]. While these results are in line with several other studies, the relative proportion of infiltrating Mo changes depending on the inflammatory context (14, 19, 25). Contrastingly, in the BM and the spleen, both NCMo and CMo reside mainly within the tissue in steady state (4.1 ± 1.4% and 3.6 ± 2.1% CD45^+^ cells in BM and spleen, respectively) as well as 4 h after LPS inoculation with a higher proportion in BM vasculature when compared to steady state (21.9 ± 1.6% 4.6 ± 0.3% CD45^+^ cells in BM and spleen, respectively; Figures 3C,D). NCMo were originally defined as patrolling Mo for their ability to crawl on the luminal side of the endothelium (17). With these last observations, patrolling denomination should be carefully used and considered as a distinct subset among NCMo that are not exclusively intravascular depending on the tissue. It is likely that CD43^+^ tissue resident and CD43^+^ vascular Mo should be considered as distinct subsets across the different organs.

In conclusion, we show that CMo express higher level of CX3CR1than NCMo hence the definition of these subsets as CX3CR1^low^ and CX3CR1^high^, respectively, should not be used in mice. In the absence of EGFP reporter, we propose to refer to an alternative phenotypic strategy using Ly6C and CD43 to identify Mo subsets at homeostasis and inflammation, in blood and tissues.

## Data Availability Statement

The raw data supporting the conclusions of this article will be made available by the authors, without undue reservation, to any qualified researcher.

## Ethics Statement

The animal study was reviewed and approved by Comité d'éthique en expérimentation animale Charles Darwin N°5.

## Author's Note

This manuscript has been released as a pre-print at bioRxiv, Meghraoui-Kheddar et al. (26).

## Author Contributions

AM-K, AB, and CC designed the study and wrote the manuscript. AM-K performed experimental work, compiled the data, and performed data analysis. SB performed the genotyping of the mice. AB and CC co-authored the manuscript and provided financial support. All authors contributed in reviewing the manuscript.

## Conflict of Interest

The authors declare that the research was conducted in the absence of any commercial or financial relationships that could be construed as a potential conflict of interest.

## Supplementary Material

Supplemental Figure 1 t-SNE was used to computationally arrange circulating immune cells according to their phenotypic similarities before (H0) and after (H4) LPS injection. Relative expression of each marker listed in Panel-1. (Supplemental Table 1) is presented in a color scale going from blue to red **(A)**. Monocytes (purple cluster), Granulocytes (red cluster), DC and B lymphocytes (green cluster), T lymphocytes (orange cluster), and NK cells (blue cluster) were hand gated **(B)** on the basis of these markers expression.

Supplemental Figure 2 Representative histogram plots of Ly6C, CCR2, CD36, CD62L, CD11b, CD64, CD43, MHCII markers, and *Nr4a1* reporter expression on CMo (blue), IMo (orange), NCMo (green) at H0 and H4 after LPS injection are presented.

Supplemental Table 1 Antibodies panel list.
