# Supplementary figures and images for: Revising CX3CR1 Expression on Murine Classical and Non-classical Monocytes
Source: Front Immunol. 2020 Jun 3;11:1117. doi: 10.3389/fimmu.2020.01117 (PMC7283740; doi:10.3389/fimmu.2020.01117)

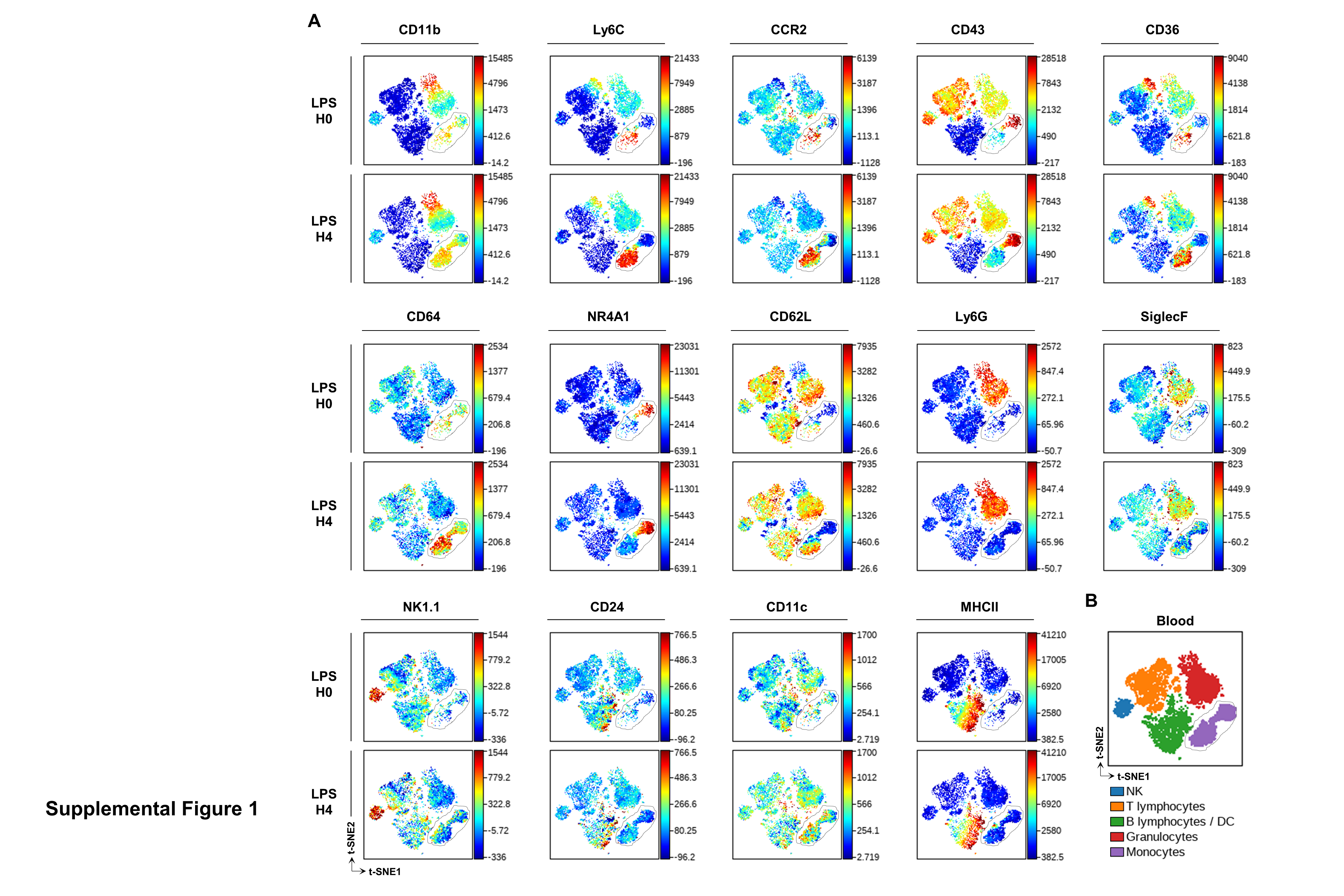

Supplement: Supplemental Figure 1 — t-SNE was used to computationally arrange circulating immune cells according to their phenotypic similarities before (H0) and after (H4) LPS injection. Relative expression of each marker listed in Panel-1. (Supplemental Table 1) is presented in a color scale going from blue to red (A). Monocytes (purple cluster), Granulocytes (red cluster), DC and B lymphocytes (green cluster), T lymphocytes (orange cluster), and NK cells (blue cluster) were hand gated (B) on the basis of these markers expression. [file Image_1.tif]

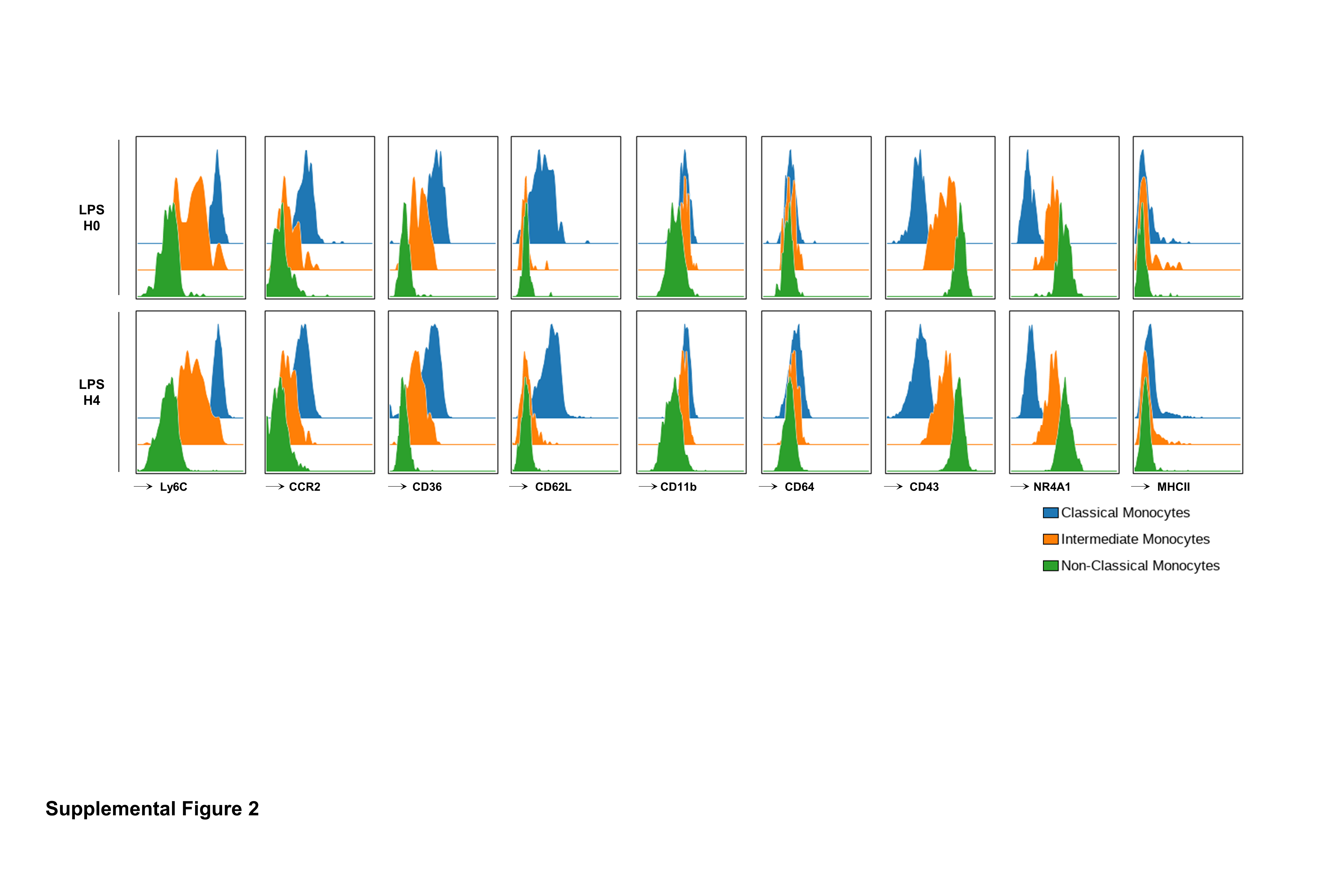

Supplement: Supplemental Figure 2 — Representative histogram plots of Ly6C, CCR2, CD36, CD62L, CD11b, CD64, CD43, MHCII markers, and Nr4a1 reporter expression on CMo (blue), IMo (orange), NCMo (green) at H0 and H4 after LPS injection are presented. [file Image_2.tif]
