# Supplementary material for: Revising CX3CR1 Expression on Murine Classical and Non-classical Monocytes
Source: Front Immunol. 2020 Jun 3;11:1117. doi: 10.3389/fimmu.2020.01117 (PMC7283740; doi:10.3389/fimmu.2020.01117)
Supplement: Supplemental Table 1 — Antibodies panel list. [file Table_1.pdf]

Fluorochrome	Panel-1			Panel-2		
	**Marker**	**Clone**	**Provider**	**Marker**	**Clone**	**Provider**
PE	CX3CR1	SA011F11	Biolegend	CX3CR1*	SA011F11	Biolegend
PECF594	SiglecF	E50-2440	BD	Ly6G	1A8	BD
PECy7	CD64	PC61	Biolegend	/	/	/
PerCPCy5.5	ivCD45	30-F11	BD	/	/	/
APC	CD36	CRF D-2712	BD	CX3CL1-AF647*	/	Almac
APCCy7	Ly6C	AL-21	BD	Ly6C	AL-21	BD
BV421	CCR2	SA203G11	Biolegend	CCR2	SA203G11	Biolegend
BV510	MHCII	M5/114	BD	SiglecF	E50-2440	BD
BV605	CD11c	E50-2440	BD	/	/	/
BV711	NK1.1	PK136	BD	NK1.1	PK136	BD
BV786	CD43	S7	BD	CD43	S7	BD
BUV395	CD11b	M1/70	BD	CD11b	M1/70	BD
BUV496	CD24	M1/69	BD	CD3	145-2C11	BD
BUV563	Ly6G	1A8	BD	/	/	/
BUV737	CD62L	MEL-14	BD	/	/	/

* In panel 2, either CX3CL1-AF647 or anti-CX3CR1-PE were used in parallel staining of the same samples

Tab. 1: Antibodies panels list
